# Serum Endocan Levels as a Risk Factor for Peripheral Artery Disease in Non-Dialysis Patients with Chronic Kidney Disease Stages 3–5

**DOI:** 10.3390/medicina61040577

**Published:** 2025-03-24

**Authors:** Kai-Jen Cheng, Hsiao-Teng Chang, Yahn-Bor Chern, Chun-Feng Wu, Jen-Pi Tsai, Bang-Gee Hsu

**Affiliations:** 1 Department of Biological Sciences, National Sun Yat-Sen University, Kaohsiung 80424, Taiwan; 2 Division of Nephrology, Hualien Tzu Chi Hospital, Buddhist Tzu Chi Medical Foundation, Hualien 97004, Taiwan; 3 Division of Nephrology, Department of Internal Medicine, Yuan’s General Hospital, Kaohsiung 80249, Taiwan; 4 Division of Metabolism and Endocrinology, Dalin Tzu Chi Hospital, Buddhist Tzu Chi Medical Foundation, Chiayi 62247, Taiwan; 5 School of Medicine, Tzu Chi University, Hualien 97004, Taiwan; 6 Division of Nephrology, Department of Internal Medicine, Dalin Tzu Chi Hospital, Buddhist Tzu Chi Medical Foundation, Chiayi 62247, Taiwan

**Keywords:** chronic kidney disease, endocan, peripheral arterial disease, ankle–brachial index, diabetes mellitus

## Abstract

*Background and Objectives*: Peripheral arterial disease (PAD) is a severe manifestation of atherosclerosis that disproportionately affects patients with chronic kidney disease (CKD) stages 3–5, resulting in a higher prevalence in this group. Currently, it is challenging to detect early PAD in this patient population. This study investigated the association between serum endocan levels and PAD based on the ankle–brachial index (ABI) in non-dialysis patients with CKD stages 3–5. *Materials and Methods*: Specimens of blood and baseline demographic characteristics were gathered from a total of 164 patients presenting with stages 3–5 CKD, who were not receiving dialysis. We used a commercially available oscillometric technique to ascertain ABI values for our participants, and used a common and well-established threshold for defining low ABI, known to be associated with PAD: ABI values < 0.9. Endocan levels in patients’ serum samples were measured by using enzyme-linked immunosorbent assays. *Results*: A total of 24 out of 164 people (14.6%) showed lower-than-normal ABIs. Compared to the group with normal ABIs, the individuals with low ABIs had more of the following conditions: diabetes mellitus (DM, *p* = 0.030), urine protein-to-creatinine ratio (*p* = 0.031), serum C-reactive protein concentrations (*p* = 0.037), and serum endocan levels (*p* < 0.001). After adjusting for variables significantly correlated with PAD by multivariate logistic regression analysis, age (odds ratio [OR]: 1.097, 95% confidence interval [CI]: 1.038–1.159, *p* = 0.001), DM (OR: 3.437, 95% CI: 1.053–11.225, *p* = 0.041), and serum endocan concentration (OR: 1.098, 95% CI: 1.042–1.157, *p* = 0.001) were identified as independent predictors of PAD in patients with CKD stages 3–5. *Conclusions*: Elevated serum endocan levels were found to be independent correlates of PAD in non-dialysis patients with CKD stages 3 through 5.

## 1. Introduction

Peripheral arterial disease (PAD) is late-stage atherosclerotic end-organ damage that targets systemic arteries and ultimately destroys blood vessels. The prevalence of PAD is striking among people who have chronic kidney disease (CKD). Rates range from 17% to 48%, a figure that far exceeds that of the general population, which stands at 5.9% [1]. The pathophysiological association between PAD and CKD is bidirectional and influenced by traditional and nontraditional risk factors [2,3,4]. The high prevalence of PAD in patients with CKD may result in serious outcomes, including pain and claudication, non-healing wounds, and critical limb ischemia, which affect the patient’s quality of life and increase their morbidity and mortality risks [5,6,7,8].

Currently, the initial screening and diagnostic modality for PAD is the resting ankle–brachial index (ABI) [9]. However, using ABI to diagnose PAD in patients with CKD has limitations, such as having a value of >1.4 due to the unique pathogenic process of vascular calcifications caused by chronic inflammation, oxidative stress, and CKD-induced mineral bone disease [10,11]. In addition, using ABI alone may have limited power in diagnosing early endothelial changes or subclinical atherosclerosis. Other tools, such as angiography, carry an additional risk of contrast-induced nephropathy in this population. These challenges underscore the importance of developing comprehensive screening tools and protocols to detect PAD early in patients with CKD.

Endocan is also known as endothelial cell-specific molecule-1 in the literature. It is a soluble proteoglycan which is primarily found within the vascular endothelial cells of humans’ pulmonary and renal systems. This biomolecule has the capacity to be released into the bloodstream in response to a variety of inflammatory stimuli [12]. Moreover, endocan levels are reportedly higher in patients with hypertension, diabetes mellitus (DM), CKD, malignancy, and cardiovascular diseases according to the proposed mechanism of inflammation and endothelial dysfunction [13,14,15,16]. However, the specific relationship between serum endocan levels and PAD remains unexplored in non-dialysis patients with CKD stages 3–5.

This study aimed to clarify the role of endocan as a serum marker for PAD using ABI examinations in patients with CKD. The rationale behind this is that since endothelial injury is viewed as the critical part of an atherosclerotic lesion, endocan, as an endothelial marker, could have a role in the initiation and development of atherosclerotic cardiovascular diseases, including PAD. Furthermore, based on our current understanding of various biological roles and conditions related to elevated endocan concentration, which overlap with many risk factors contributing to PAD, such as DM, CKD, and hypertension, our study could contribute novel insights into the early detection and risk stratification of PAD in patients with renal insufficiency.

## 2. Materials and Methods

### 2.1. Participants

We enrolled 164 individuals diagnosed with CKD at stages 3 to 5 from a medical center in Hualien, Taiwan, during the period spanning from January to August 2020. CKD was diagnosed based on two separate estimated glomerular filtration rate (eGFR) values as determined by the Chronic Kidney Disease Epidemiology Collaboration equation, with measurements taken at intervals of >3 months. When the eGFR is less than 60 mL/min/1.73 m^2^, the patient has CKD, and we then use the criteria established by the Kidney Disease Outcomes Quality Initiative to sort the patient into stages as follows: stage 3 (eGFR ranging from 59 to 30 mL/min/1.73 m^2^), stage 4 (eGFR ranging from 29 to 15 mL/min/1.73 m^2^), and stage 5 (eGFR below 15 mL/min/1.73 m^2^) [17]. We excluded patients on maintenance dialysis, with acute infections, malignancies, strokes, amputations, or heart failure, treated with cilostazol or pentoxifylline during blood sampling, and those with an ABI > 1.3, as shown in Table 1.

Blood pressure (BP) readings were taken in the morning by qualified personnel after the patients had rested for a minimum of 10 min. A standardized, mercury sphygmomanometer of an appropriate size was used to secure the readings. The average reading was taken from three sequential measurements. These measurements were made in a consistent manner, at 5 min intervals, and under similar environmental conditions. The BP data were then evaluated. If a person’s BP was equal to or greater than 140/90 mm Hg, or if he or she had taken antihypertensive therapy anytime within the preceding 2 weeks, that person’s condition was classified as hypertension. Persons determined to have diabetes mellitus (DM) were identified through fasting plasma glucose concentrations of ≥126 mg/dL or through treatment with one or more antidiabetic agents.

The approval for this investigation was granted by the Institutional Review Board of Tzu Chi Hospital (IRB108-219-A). All participants involved provided their informed consent, which was documented in writing.

### 2.2. Anthropometric Analysis

Anthropometric parameters were collected in the morning after an overnight fasting period. Weights and measures of physical stature were recorded and rounded to the nearest 0.5 kg and 0.5 cm, respectively. The body mass index (BMI) was determined by dividing the weight (kg) by the square of the height (m^2^).

### 2.3. Biochemical Investigations

Fasting blood samples (approximately 5 mL) were collected from each patient. The remaining sample was centrifuged for biochemical analyses after determining the hemoglobin level (Sysmex SP-1000i, Sysmex American, Mundelein, IL, USA). Parameters measured included blood urea nitrogen (BUN), creatinine, albumin, total cholesterol, triglycerides (TG), low-density lipoprotein cholesterol, fasting glucose, and C-reactive protein (CRP). Additionally, the urine protein-to-creatinine ratio (UPCR) was evaluated through random spot urine testing using an autoanalyzer (SiemensAdvia 1800, Siemens Healthcare GmbH, Henkestr, Germany). Serum levels of human endocan were measured using commercially available enzyme-linked immunosorbent assays (Aviscera Bioscience, Inc., Santa Clara, CA, USA).

### 2.4. Blood Pressure and ABI Measurements

BP was measured three times and taken from both the upper and lower extremities over the arms and ankles. The person was placed in a supine position. BP readings were acquired from the brachial, dorsalis pedis, and posterior tibial arteries. The oscillometric technique was used for assessing BP, and the measurement apparatus was the VaSera VS-1000 (Fukuda Denshi Co, Ltd.; Tokyo, Japan). The ABI was determined as the quotient of the maximum systolic BP recorded in either the right or left ankle (dorsalis pedis or posterior tibial artery) relative to the maximum systolic BP recorded in either brachial artery. Continuous electrocardiographic monitoring was conducted for a duration exceeding 15 min. PAD was diagnosed in patients with a measured ABI of less than 0.9 in either the left or right limb, based on established diagnostic criteria. All of the above procedures were performed by the same trained staff.

### 2.5. Statistical Analysis

The result of the power analysis for this study was as follows: to detect a correlation coefficient of about 0.3 between serum endocan levels and ABI, with an alpha level of 0.05 and a power of 80%, a total of at least 85 patients should be included in the study, and to detect a correlation coefficient of about 0.3 between serum endocan levels and ABI, with an alpha level of 0.05 and a power of 90%, a total of at least 113 patients should be included in the study.

The Kolmogorov–Smirnov test is used to assess the normality of continuous data. Variables showing a normal distribution are delineated as means ± standard deviation, while those that do not conform to a normal distribution (specifically left and right ABI, TG, fasting glucose, BUN, creatinine, UPCR, CRP, and endocan levels) are represented as medians accompanied by interquartile ranges. Between-group comparisons were conducted using a two-tailed Student’s independent t-test for normally distributed data and the Mann–Whitney *U* test for non-normally distributed data. Categorical variables were summarized as numbers and percentages, and comparisons were made using the χ^2^ test. Variables significantly associated with PAD (*p* < 0.05 in univariate analysis) were included into the multivariate logistic regression model to identify independent predictors. These variables were age, diabetes mellitus, CRP, UPCR, and serum endocan levels. The area under the curve (AUC) was calculated using receiver operating characteristic (ROC) curve analysis to assess the predictive ability of serum endocan levels for diagnosing PAD in non-dialysis patients with CKD stages 3–5. Non-normally distributed variables underwent base 10 logarithmic transformations to achieve normality. The association between log-endocan, left log-ABI, right log-ABI measurements, and clinical variables was evaluated using Spearman’s rank correlation coefficient. All statistical evaluations were conducted utilizing the SPSS software for Windows (version 19.0; SPSS, Chicago, IL, USA). A *p*-value of less than 0.05 was deemed statistically significant. 

## 3. Results

The baseline characteristics of the 164 patients (control [*n* = 140] and low ABI [*n* = 24]) are shown in Table 2. The prevalence of DM, hypertension, and chronic glomerulonephritis was 31.1% (*n* = 51), 81.7% (*n* = 134), and 15.2% (*n* = 25), respectively. Furthermore, 13.4% (*n* = 22) of the patients were current smokers. The distribution of gender, BMI, serum lipid levels, BUN, creatinine levels, eGFR, albumin concentration, hemoglobin levels, CKD stage, or concurrent glomerulonephritis were not significantly different in the two cohorts. In addition, there was no statistically significant differences between these two groups regarding the prescriptions of anti-hypertensive agents, such as angiotensin II receptor antagonists, beta-adrenergic blockers, calcium channel antagonists, and lipid-lowering agents, including statins or fibrates. Some significant results were found when comparing the control group with people in the low-ABI group. Firstly, people in the low-ABI group were significantly older (*p* < 0.001), and they also had more DM and elevated levels of UPCR (*p* = 0.031) than the people in the control cohort (*p* = 0.030). Two other findings were that people in the low-ABI group had elevated levels of CRP (*p* = 0.027) and higher levels of serum endocan (*p* < 0.001).

In multivariate logistic regression analysis pertaining to factors associated with PAD, including DM, age, UPCR, CRP, and endocan, we identified that elevated serum levels of endocan (odds ratio [OR]: 1.098; 95% confidence interval [CI]: 1.042–1.157; *p* = 0.001), advanced age (OR: 1.097; 95% CI: 1.038–1.159; *p* = 0.001), and the presence of DM (OR: 3.437; 95% CI: 1.053–11.225; *p* = 0.041) were independent predictors of PAD in individuals with CKD at stages 3 through 5 who are not undergoing dialysis (Table 3). 

**Figure 1:**
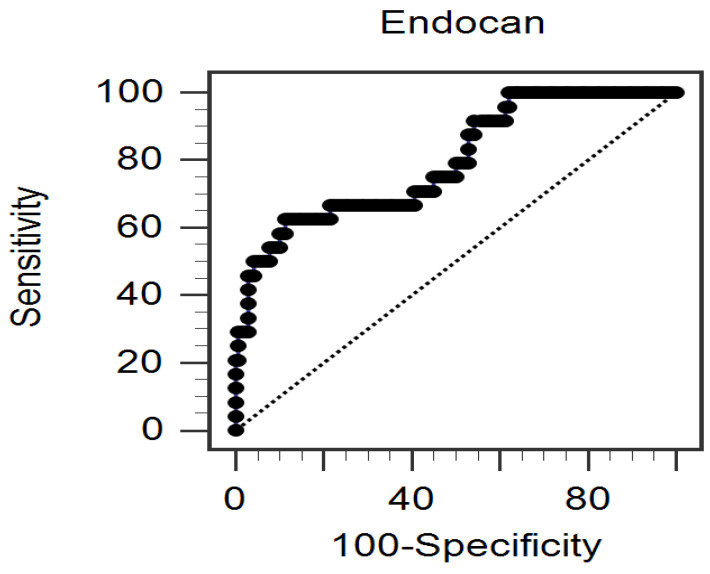
The area under the receiver operating characteristic curve was calculated to assess the power of serum endocan concentrations in predicting peripheral arterial disease. The cohort consisted of 164 individuals diagnosed with chronic kidney disease stages 3 to 5.

Table 4 shows the correlation between serum log-endocan and various clinical parameters by Spearman’s correlation analysis. The left and right log-ABI values were negatively correlated with log-endocan (*r* = −0.347 and *r* = −0.320, *p* < 0.001, respectively). Additionally, serum log-endocan showed a positive correlation with log-triglyceride (*r* = 0.157, *p* = 0.045) and log-CRP (*r* = 0.195, *p* = 0.012). Left and right log-ABI values were also negatively correlated with age (*r* = −0.401 and *r* = −0.367, *p* < 0.001, respectively) and log-CRP (*r* = −0.295 and *r* = −0.312, *p* < 0.001, respectively). Furthermore, the ROC curve for predicting PAD showed that the AUC for serum endocan was 0.797 (95% CI: 0.696–0.898, *p* < 0.0001; cut-off = 27.17 ng/mL, sensitivity = 62.50%, specificity = 88.57%, positive predictive value = 48.38%, and negative predictive value = 93.24%) (Figure 1).

## 4. Discussion

In this study, patients in the low-ABI group were older, had a higher prevalence of DM, and had significantly higher serum endocan levels. Meanwhile, the two groups had no differences in other biochemical parameters, underlying diseases, or current medication use. The finding that older age and DM were independently correlated with the presence of PAD is aligned with the current consensus regarding traditional atherosclerotic risk factors [18,19,20].

CKD is a major global health concern due to its association with an increased risk of developing cardiovascular (CV) diseases, including PAD [1]. The unique pathogenic processes of CKD, such as chronic inflammation and vascular calcification, contribute to the development of PAD in these patients [8,21]. Regarding screening for PAD in patients with CKD, current guidelines may cause delays in its diagnosis. The United States Preventive Services Task Force stated that there was insufficient evidence to screen for PAD in asymptomatic adults, while the AHA/ACC guidelines recommend screening for asymptomatic PAD only in adults with an increased risk, which does not include CKD; these guidelines may not adequately capture PAD in the high-risk CKD population [9,22]. However, the AHA/ACC guidelines recommended proceeding with the toe–brachial index for diagnosing PAD if the initial ABI result is >1.4 in patients with vascular stiffness, which is common in patients with CKD [9]. Moreover, the K/DOQI guidelines suggested screening for PAD at the initiation of dialysis [23]. Due to the above factors and challenges in managing patients with PAD and vascular calcification, additional screening tools to detect PAD early in patients with CKD are warranted.

Several risk factors for atherosclerotic PAD are categorized into traditional and nontraditional risk factors. Traditional risk factors are further divided into non-modifiable and modifiable types. Meanwhile, non-modifiable risk factors include age, sex, and polygenic or family inheritance. Modifiable risk factors include tobacco use, DM, dyslipidemia, and hypertension. Nontraditional risk factors are depicted in a broader range, including female-specific factors, like pregnancy-induced hypertension or diabetes and menopause. Additional nontraditional risk factors include socioeconomic status, environmental pollution, stress, autoimmune diseases, apolipoprotein levels, inflammation, and unhealthy lifestyle choices such as being overweight, sedentary lifestyle, sleep disorders, a stressful lifestyle, alcohol consumption, and diet. Nevertheless, these factors contribute to the development and progression of atherosclerotic PAD [24]. Pan et al. explored the association between PAD and kidney function, as assessed by eGFR and albumin-to-creatinine ratio (ACR), in patients with type 2 DM and revealed that a lower eGFR was significantly associated with PAD, and that within each eGFR stage, a higher ACR category was similarly associated [25]. Patients with an eGFR of <30 mL/min/1.73 m^2^ and macroalbuminuria showed the highest odds of developing PAD (OR 14.42, 95% CI: 4.60–45.31), compared with those with an eGFR of ≥90 mL/min/1.73 m^2^ and normoalbuminuria. Using a cross-sectional analysis, Sinjari et al. evaluated the prevalence, clinical characteristics, and risk factors of PAD in 175 patients with CKD stages 3–5 in Iraq, revealing significant associations between PAD and an older age, male sex, hypertension, DM, advanced CKD stages, prior coronary artery disease, albuminuria, elevated glycated hemoglobin levels, and high-sensitivity CRP levels [26]. In this study, the number of non-dialysis patients with CKD stages 3–5 who were older, had DM, and had higher UPCR and CRP levels were significantly higher in the low-ABI group than those in the normal-ABI group. Following multivariate logistic regression analysis, traditional risk factors, such as age and DM, alongside nontraditional CKD-specific factors, such as CRP, were revealed as factors that modulate the pathogenesis of PAD in patients with CKD. Age and CRP were also negatively associated with log-ABI levels in this study.

Endocan has been investigated as a potential biomarker for several inflammation and endothelial dysfunction conditions, including PAD [14,27,28,29]. In a prospective nationwide study on patients who underwent prior percutaneous coronary intervention, higher endocan levels were independently associated with hard CV outcomes (CV mortality, non-fatal myocardial infarction, and stroke) [30]. Additionally, the authors conducted an in vitro study that showed that endocan can influence cell migration, tube formation (indicating improvement of endothelial progenitor cell function), and adhesiveness, confirming that endocan is important in vascular repair and atherosclerosis progression. On multivariate analysis, Yilmaz et al. revealed that elevated endocan levels in non-dialysis patients with CKD were significantly associated with higher high-sensitivity CRP levels, decreased flow-medicated vasodilation, and increased carotid intima–media thickness [16]. Their study also significantly associated endocan levels with CV events and all-cause mortality on COX survival analysis [16]. The correlation between endocan and atherosclerotic CV disease is also observed among patients with end-stage renal disease, with even higher levels compared to non-dialysis patients with CKD [31,32,33]. Yazman et al. assessed endocan as a biomarker for diagnosing and following PAD, revealing that endocan levels were significantly higher in the PAD group than in the control group, and decreased 1 month later following medical or surgical treatment for PAD [34]. In this study, the serum log-endocan level was negatively associated with the log-ABI level and positively associated with the log-CRP level. Following multivariate logistic regression analysis, the serum endocan level became an independent predictor of PAD in non-dialysis patients with CKD stages 3–5. Considering the unique pathogenic mechanisms of CKD and challenges in diagnosing PAD early, endocan is a promising biomarker to help clinicians better identify patients at high risk for PAD.

**Figure 2:**
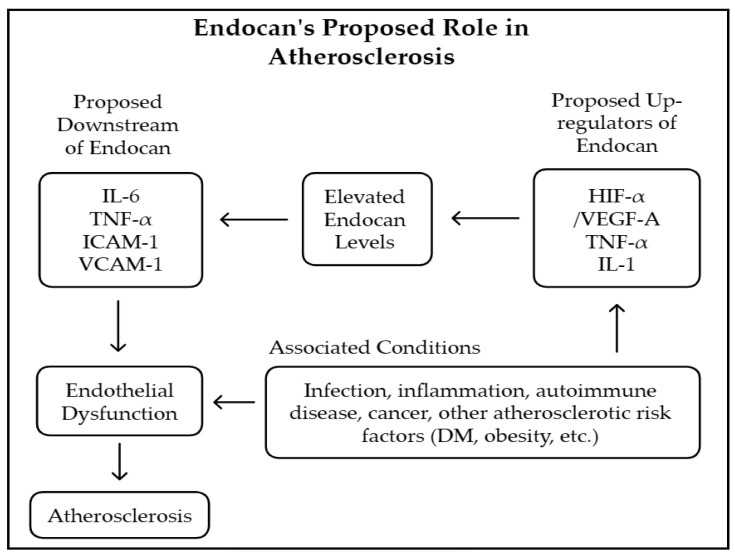
The proposed mechanistic pathways of how endocan could be associated with the initiation and progression of atherosclerosis [40]. (Abbreviations: IL, interleukin; TNF-α, tumor necrosis factor-α; ICAM-1, intercellular adhesion molecule-1; VCAM-1, vascular cell adhesion molecule-1; HIF-α, hypoxia-inducible factor-α; VEGF, vascular endothelial growth factor; DM, diabetes mellitus.).

With respect to the potential mechanistic pathways linking endocan to PAD, in which atherosclerosis is the major contributor, several studies have explored this topic. As shown in Figure 2, endocan could be up-regulated in the presence of tumor necrosis factor-α (TNF-α)-, interleukin-1 (IL-1)-, and vascular endothelial growth factor (VEGF)-mediated pathways, which are all key players in the development of inflammation and endothelial dysfunction [35,36]. Then, the downstream effects of increased endocan levels result in enhanced leukocyte adherence, activation, and proliferation through intercellular adhesion molecule-1 (ICAM-1) and vascular cell adhesion molecule-1 (VCAM-1) [31,37]. Subsequently, cytokines such as TNF-α and IL-6 are released to accomplish the inflammation cascade that impairs endothelial cell functions [38,39]. Following this, there is reduced nitric oxide (NO) and increased reactive oxygen species (ROS), contributing to the pathogenesis of atherosclerotic cardiovascular disease. Additionally, risk factors for atherosclerosis, such as diabetes, obesity, and hypertension, have been shown to alter circulating endocan levels [40]. Because of the complexity of endocan’s interactions with a variety of factors related to atherosclerosis, one might raise the question of whether endocan is a bystander or a critical player in the role of inflammation-mediated vasculopathy in PAD. However, the answer to this question requires further studies to elucidate the mechanisms that are involved.

This study provides promising prospects for enhancing clinical practices in caring for CKD patients with PAD. By establishing that elevated serum endocan levels are independently associated with lower ABI values and, hence, the presence of PAD, this research underscores the potential of endocan as a non-invasive biomarker for early detection. One potential clinical implication is bringing serum endocan measurement into everyday clinical use—possibly along with other biomarkers and risk stratification scores, as well as screening tools, such as ABI or toe–brachial index (TBI)—to achieve risk stratification that is better than what we currently have, and that allows us to identify, before they become symptomatic, the patients who are most likely to progress in an adverse direction. Previous study findings have shown that traditional screening tools for PAD, such as ABI or TBI, revealed low sensitivity in CKD patients. This is caused by medial arterial calcification, a common pathology in CKD, leading to pseudo-normalization of ABI or TBI values [41]. Therefore, adding endocan assessment to the diagnostic workup of PAD in CKD stages 3–5 may provide incremental value in identifying this high-risk population. Another well-known inflammatory marker, CRP, has demonstrated an association with PAD [42]. However, CRP is a non-specific marker of inflammation, and the inclusion of endocan, which is more directly linked to endothelial dysfunction, may improve risk stratification for PAD in this population. Diagnosing PAD early permits the clinician to intervene in a timely manner, and it may also assist in reducing the overall atherosclerotic burden in people with CKD. This could enhance long-term cardiovascular results and diminish complications like critical limb ischemia. In conclusion, the biomarker-driven approach could enable a shift toward more personalized management strategies for this population, with less reliance on invasive diagnostic or operator dependent modalities—a change that could have a major impact on the quality of life of these patients.

This study has several limitations. First, this study did not include patients with CKD on maintenance dialysis, who typically have higher serum endocan levels. Consequently, a comparison between non-dialysis and dialysis groups was not conducted. Second, the limited number of patients from only one institution may limit the generalizability of the results. Additional cases from various settings would strengthen the relationship between serum endocan levels and PAD. Third, atherosclerosis is a natural consequence of aging, and PAD is prevalent in elderly individuals, regardless of the presence of DM. Given the advanced age of the study population, particularly in the low-ABI group, further investigations involving younger patients are necessary to reveal the relationship between serum endocan levels and PAD in different age groups. Another limitation is that our study excluded patients with an ABI >1.3, primarily due to the lack of appropriate diagnostic facilities for such cases. This exclusion may overlook a subset of patients with CKD with PAD, as PAD with elevated ABI values is not uncommon in this population. Finally, further studies are required to clarify the impact of confounding factors on the endocan concentration, such as coexisting comorbidities, medication effects, inflammatory conditions, and lifestyle or socioeconomic factors.

Based on the insights gained from this study, several future research directions must be considered. First, additional research recruiting patients of diverse ethnic and geographic backgrounds can improve the external validity of our findings. In addition, as endothelial biomarkers could be affected by various genetic and environmental conditions, a multi-center design of study is required to determine whether the association of endocan and PAD can be consistently demonstrated across different populations. Next, since endothelial function and inflammatory status are influenced by dialysis, it would be valuable to compare between non-dialytic and dialytic individuals to evaluate the prognostic significance of endocan in patients with more advanced CKD. Thirdly, it is important to perform longitudinal studies to elucidate the relationship between serum endocan levels, PAD progression, and total cardiovascular risk. Another potential direction for future research is that with more studies regarding the mechanisms of endocan in endothelial dysfunction, vascular calcification, and repair processes, we might learn much more about the initiation and development of atherosclerosis that occurs in CKD. There is also potential in looking to see whether therapeutic interventions that modify endocan levels might result in some clinical benefits—better vascular function, for instance, or fewer cardiovascular events. Ultimately, the integration of endocan with other biomarkers or risk stratification scores holds great potential for the development of a novel risk assessment model. This could provide a helpful guide in precision medicine approaches to the management of CKD patients.

## 5. Conclusions

Our study revealed a significant association between increased endocan levels and PAD, as demonstrated by the low ABIs in patients with CKD stages 3–5. Furthermore, we found that DM and older age are independent predictors of PAD in this population. As a result of the increasing prevalence of these risk factors in both the general population and CKD patients, there is a rising demand for more effective and reliable screening and diagnosing tools for PAD.

The findings of our study revealed that endocan could be a novel biomarker of endothelial dysfunction and inflammation. Endocan may have benefits compared to traditional biomarkers such as CRP and ABI, which have noticeable limitations because of the nature of CKD and its effects on the blood vessels. Blood vessel calcification and stiffening, which happen in CKD, markedly impair the detection of PAD through standard means. However, measuring endocan could facilitate timely therapeutic interventions, personalized risk stratification, and improved cardiovascular outcomes in CKD patients.

Future research should focus on validating endocan’s role as a PAD biomarker in larger, multi-center cohorts, assessing its predictive value in different CKD stages, and exploring its integration into current clinical guidelines. Additionally, it is important to conduct longitudinal studies to see if monitoring endocan levels over time can help us track disease progression and evaluate treatment response. After obtaining a better understanding of the link between endothelial dysfunction and PAD, we can develop better ways to screen patients and create more effective treatments for CKD patients who are at a higher risk for atherosclerotic cardiovascular diseases.

## Figures and Tables

**Table 1:** The inclusion and exclusion criteria of the study.

Inclusion Criteria	Exclusion Criteria
CKD stages 3 to 5	Dialysis
Adults (not less than 18 years old)	Acute infection
	Malignancy
	Stroke
	Heart failure
	Under cilostazol or pentoxifylline
	ABI > 1.3

Abbreviations: CKD, chronic kidney disease; ABI, ankle–brachial index.

**Table 2:** Clinical variables of the 164 patients with chronic kidney disease stages 3–5 with ankle–brachial index < 0.9 or ankle–brachial index ≥ 0.9 group.

Characteristics	All Patients (*n* = 164)	Control Group (*n* = 140)	Low-ABI Group (*n* = 24)	*p* Value
Age (years)	68.48 ± 13.37	66.06 ± 12.55	80.54 ± 11.33	<0.001 *
Body mass index (kg/m^2^)	26.35 ± 4.80	26.58 ± 4.58	24.98 ± 5.85	0.132
Left-ankle–brachial index	1.07 (0.99–1.13)	1.08 (1.03–1.14)	0.77 (0.66–0.85)	<0.001 *
Right-ankle–brachial index	1.09 (1.02–1.14)	1.10 (1.05–1.14)	0.78 (0.67–0.87)	<0.001 *
Hemoglobin (g/dL)	11.67 ± 2.48	11.78 ± 2.57	11.04 ± 1.77	0.179
Total cholesterol (mg/dL)	159.11 ± 33.53	158.71 ± 33.71	161.46 ± 30.03	0.712
Triglyceride (mg/dL)	119.00 (87.25–164.00)	119.00 (86.25–163.50)	117.50 (91.75–166.25)	0.867
LDL-C (mg/dL)	91.45 ± 29.46	90.67 ± 28.35	96.00 ± 33.82	0.410
Fasting glucose (mg/dL)	104.50 (96.00–133.00)	101.50 (96.00–130.75)	109.50 (95.00–142.50)	0.310
Albumin (g/dL)	4.04 ± 0.33	4.06 ± 0.34	3.95 ± 0.26	0.145
Blood urea nitrogen (mg/dL)	35.50 (26.00–48.00)	38.00 (26.00–49.00)	32.00 (26.00–38.75)	0.153
Creatinine (mg/dL)	2.00 (1.50–2.78)	2.10 (1.50–2.98)	1.80 (1.40–2.28)	0.117
eGFR (mL/min)	30.42 ± 14.51	30.26 ± 15.07	31.38 ± 10.82	0.729
UPCR (mg/g)	182.00 (71.55–453.00)	131.00 (61.95–475.75)	266.80 (187.50–344.75)	0.031 *
CRP (mg/dL)	0.07 (0.05–0.64)	0.05 (0.05–0.47)	0.56 (0.05–2.64)	0.027 *
Endocan (ng/mL)	15.75 (9.30–24.12)	14.74 (8.73–21.71)	29.42 (15.08–46.07)	<0.001 *
Female, *n* (%)	67 (40.9)	57 (40.7)	10 (41.7)	0.930
Diabetes mellitus, *n* (%)	51 (31.1)	39 (27.9)	12 (50.0)	0.030 *
Hypertension, *n* (%)	134 (81.7)	114 (81.4)	20 (83.3)	0.824
Glomerulonephritis, *n* (%)	25 (15.2)	21 (15.0)	4 (16.7)	0.834
Current smoking, *n* (%)	22 (13.4)	18 (12.9)	4 (16.7)	0.613
CKD stage 3, *n* (%)	76 (46.3)	63 (45.0)	13 (54.2)	0.322
CKD stage 4, *n* (%)	56 (34.1)	47 (33.6)	9 (37.5)	
CKD stage 5, *n* (%)	32 (19.5)	30 (21.4)	2 (8.3)	

For continuous variables, the measurements are expressed as mean ± standard deviation and processed using Student's *t*-test for tests of significance. If a variable does not seem to follow a normal distribution, it is expressed as a median and interquartile range, and the Mann–Whitney *U* test is used for significance testing. The chi-square test is used to determine if there is a significant association between categorical variables, which are shown as frequency (%). (Abbreviations: ABI, ankle–brachial index; LDL-C, low-density lipoprotein cholesterol; eGFR, estimated glomerular filtration rate; UPCR, urine protein-to-creatinine ratio; CRP, C-reactive protein; CKD, chronic kidney disease.) * *p* < 0.05 was considered statistically significant.

**Table 3:** A multivariate logistic regression analysis examining the factors associated with peripheral arterial disease in a cohort of 164 individuals diagnosed with chronic kidney disease stages 3 through 5.

Variables	Odds Ratio	95% Confidence Interval	*p* Value
Endocan, 1 ng/mL	1.098	1.042–1.157	0.001 *
Age, 1 year	1.097	1.038–1.159	0.001 *
Diabetes mellitus, present	3.437	1.053–11.225	0.041 *
C-reactive protein, 1 mg/dL	1.119	0.816–1.535	0.486
UPCR, 1 mg/g	1.000	0.999–1.001	0.795

UPCR, urine protein-to-creatinine ratio. The dataset underwent examination via multivariate logistic regression analysis, incorporating variables such as diabetes mellitus, chronological age, C-reactive protein levels, urinary protein-to-creatinine ratio, and endocan concentration. * *p* < 0.05 was considered statistically significant.

**Table 4:** The Spearman correlation coefficients calculated between the left ABI, right ABI, log-transformed endocan levels, and various clinical variables in a cohort of 164 individuals diagnosed with chronic kidney disease.

Variables	Left Log-ABI		Right Log-ABI		Log-Endocan (ng/mL)	
	Spearman Coefficient of Correlation	*p* Value	Spearman Coefficient of Correlation	*p* Value	Spearman Coefficient of Correlation	*p* Value
Age (years)	–0.401	<0.001 *	–0.367	<0.001 *	0.129	0.099
Body mass index (kg/m^2^)	0.127	0.105	0.142	0.070	0.077	0.329
Left log-ABI	**—**	**—**	0.905	<0.001 *	–0.347	<0.001 *
Right log-ABI	0.905	<0.001 *	**—**	**—**	–0.320	<0.001 *
Log-endocan (ng/mL)	–0.347	<0.001 *	–0.320	<0.001 *	**—**	**—**
SBP (mmHg)	–0.117	0.137	–0.146	0.063	–0.055	0.483
DBP (mmHg)	0.122	0.120	0.124	0.113	–0.152	0.053
Hemoglobin (g/dL)	0.080	0.308	0.048	0.544	–0.013	0.871
Total cholesterol (mg/dL)	–0.055	0.484	–0.074	0.349	0.116	0.138
Log-triglyceride (mg/dL)	–0.017	0.832	–0.035	0.659	0.157	0.045 *
LDL-C (mg/dL)	–0.052	0.510	–0.083	0.289	0.136	0.082
Log-glucose (mg/dL)	–0.042	0.592	–0.091	0.245	0.116	0.140
Albumin (g/dL)	0.125	0.112	0.106	0.177	0.024	0.763
Log-BUN (mg/dL)	0.062	0.432	0.063	0.420	–0.108	0.167
Log-creatinine (mg/dL)	0.142	0.070	0.136	0.082	–0.096	0.222
eGFR (mL/min)	0.006	0.943	–0.013	0.865	0.080	0.309
Log-UPCR (mg/g)	–0.117	0.135	–0.115	0.144	–0.016	0.844
Log-CRP (mg/L)	–0.295	<0.001 *	–0.312	<0.001 *	0.195	0.012 *

Data including ABI, triglyceride levels, glucose concentrations, BUN, creatinine measurements, UPCR, CRP, and levels of endocan revealed a skewed distributed pattern and were logarithmically transformed before statistical analysis. Spearman correlation coefficients were applied with respect to the analysis of the data. Abbreviations: ABI, ankle–brachial index; LDL-C, low-density lipoprotein cholesterol; BUN, blood urea nitrogen; SBP, systolic blood pressure; DBP, diastolic blood pressure; eGFR, estimated glomerular filtration rate; UPCR, urine protein-to-creatinine ratio; and CRP corresponds to C-reactive protein. * *p* < 0.05 was regarded as statistically significant (two-tailed).

## Data Availability

Upon request, the corresponding author can provide the data utilized in this study.
